# Radiation-Induced Bystander Effect is Mediated by Mitochondrial DNA in Exosome-Like Vesicles

**DOI:** 10.1038/s41598-019-45669-z

**Published:** 2019-06-24

**Authors:** Kentaro Ariyoshi, Tomisato Miura, Kosuke Kasai, Yohei Fujishima, Akifumi Nakata, Mitsuaki Yoshida

**Affiliations:** 1 Department of Radiation Biology, Institute of Radiation Emergency Medicine, Hirosaki University, 66-1 Hon-cho, Hirosaki, 036-8564 Japan; 2 Department of Biomedical Sciences, Hirosaki University Graduate School of Health Sciences, 66-1 Hon-cho, Hirosaki, 036-8564 Japan; 3 Department of Basic Pharmacy, Hokkaido Pharmaceutical University School of Pharmacy, Maeda 7-jo 15-4-1, Teine-ku, Otaru, Sapporo 006-8590 Japan

**Keywords:** Double-strand DNA breaks, Extracellular signalling molecules, DNA damage response

## Abstract

Exosome-like vesicles (ELV) are involved in mediating radiation-induced bystander effect (RIBE). Here, we used ELV from control cell conditioned medium (CCCM) and from 4 Gy of X-ray irradiated cell conditioned medium (ICCM), which has been used to culture normal human fibroblast cells to examine the possibility of ELV mediating RIBE signals. We investigated whether ELV from 4 Gy irradiated mouse serum mediate RIBE signals. Induction of DNA damage was observed in cells that were treated with ICCM ELV and ELV from 4 Gy irradiated mouse serum. In addition, we treated CCCM ELV and ICCM ELV with RNases, DNases, and proteinases to determine which component of ELV is responsible for RIBE. Induction of DNA damage by ICCM ELV was not observed after treatment with DNases. After treatment, DNA damages were not induced in CCCM ELV or ICCM ELV from mitochondria depleted (ρ0) normal human fibroblast cells. Further, we found significant increase in mitochondrial DNA (mtDNA) in ICCM ELV and ELV from 4 Gy irradiated mouse serum. ELV carrying amplified mtDNA (ND1, ND5) induced DNA damage in treated cells. These data suggest that the secretion of mtDNA through exosomes is involved in mediating RIBE signals.

## Introduction

The radiation-induced bystander effect (RIBE) was first reported as the phenomenon which involves the induction of sister chromatid exchanges (SCE) by extremely low doses of alpha particles in non-irradiated cells^1^. The responses of RIBE include inflammation and genotoxic effects such as DNA damage, chromosomal aberrations, mutations, apoptosis, genomic instability, and neoplastic transformations^2^. RIBE has been detected *in vitro*^3–6^, in 3D tissues^7^, and in mouse models^8–10^. Growing evidence shows that bystander responses can be regulated by two mechanisms—(i) gap junction intercellular communication (ii) communication of soluble factors released by irradiated cells or organs^11,12^. With respect to the soluble factors, Mothersill and Seymour first reported that the irradiated medium from human epithelial cell medium displayed a toxic effect on fibroblast cells^13^. Since then, many studies have shown that medium from non-irradiated cells can induce RIBE in various cell lines^14–16^. A large variety of candidate factors as possible mediators of RIBE (cytokines, chemokines, reactive oxygen species (ROS), NO, miRNAs etc.) suggest that complex signals by multiple mediators can mediate RIBE signals^11,12,16^. Further, several evidences suggest that plasma from irradiated animals, including humans, contains factors that are able to cause chromosomal aberrations in non-irradiated cells^17–22^. These factors, called clastogenic factors, are very similar effect to RIBE.

Exosome-like vesicles (ELV), which are secreted into the extracellular environment by most types of cells, have been investigated as mediators of intercellular communication and are involved in multiple biological processes owing to their contents such as mRNAs, miRNAs, DNA, and proteins^23^. Interestingly, it has been reported that ELV secreted from senescent cells induce senescence, pro-inflammation, stem cell dysfunction, and cancer development in the neighbouring cells^24–27^. Lehmann *et al*. reported an increase in the secretion of ELV in the extracellular environment by senescent human prostate cancer cells by clinically relevant doses of irradiation^28^. It has now been established that ELV in biological processes and signalling pathways are related to cellular senescence^29^.

Although the mechanisms underlying RIBE are still unknown, the possible mechanism of RIBE, which concerns ELV, was first reported by Albanese and Dainiak^30^. They reported that ionizing radiation increases the levels of Fas ligand (FasL), also called as the “death ligand”, exfoliated on extracellular vesicles^30^. Since then, numerous reports suggest that the ELV are involved in mediating RIBE *in vivo* and also in cultured cells^31–39^.

In this study, we used the normal human fibroblast cells (HDFn) and mouse fibroblast cells (m5S) which were cultured in exosome depleted foetal bovine serum (FBS) medium to decrease the interference of serum ELV. We first observed increased DNA damages in HDFn cells treated with ICCM ELV of HDFn. Also, m5S cells treated with ELV from irradiated mouse serum showed increased DNA damage. DNase treatment in ELV from ICCM reduced the DNA damage suggesting that DNA in ELV mediates RIBE. Hence, HDFn cells treated with CCCM ELV or ICCM ELV from mitochondria depleted (ρ0) HDFn cells showed no increase in DNA damage. We found significant increase in mitochondrial DNA (mtDNA) in ELV in condition medium and mouse serum after irradiation. ELV carrying amplified mtDNA (ND1, ND5) induced DNA damage in treated cells. Our results suggest that mtDNA in ELV are involved in mediating RIBE.

## Materials and Methods

### Ethic statement

All experiments were conducted according to the guidelines for Animal Experimentation of Hirosaki University, and the procedures were approved and monitored by the Animal Research Committee of Hirosaki University (approval number: G15001).

### Animals

Seven-week-old male ICR mice (Charles River Japan Inc.) were purchased and maintained at the Institute for Animal Experiments at Hirosaki University. Mice were housed in an autoclaved cage and maintained in rooms under a regular 12-h light, 12-h dark cycle. Mice were fed a standard laboratory animal diet (MB-1: Funabashi Farm Co., Tokyo, Japan) and were supplied water *ad libitum*.

### Cell culture and cell cycle analysis

A primary normal human dermal fibroblast (HDFn) cells (ATCC PCS-201-010) were kindly provided by Dr. Toshiya Nakamura (Hirosaki University, Aomori, Japan) and an immortalized mouse embryonic fibroblast (m5S) cells (JCRB1322) were kindly provided by Dr. Seiji Kodama (Osaka prefecture University, Osaka, Japan). The HDFn cells were cultured in Dulbeccos’s modified eagle medium (DMEM; Invitrogen) supplemented with 10% exosome-depleted foetal bovine serum (Exo-FBS, System Biosciences), 100 U/ml penicillin, and 100 μg/ml streptomycin (Invitrogen). The m5S cells were cultured in alpha-MEM supplemented with 10% Exo-FBS (System Biosciences), 100 U/ml penicillin, and 100 μg/ml streptomycin. Cells were maintained at 37 °C in a humidified atmosphere with 5% CO_2_. Cell cycle phase was measured by Muse Cell Analyzer, using a Muse Cell Cycle Assay Kit (Merck Millipore) following the manufacturer’s instructions.

### X-ray exposure

Cells or mice (8-week-old) were irradiated with using an X-ray generator (MBR-1520R-3; Hitachi Medical) with 0.5 mm aluminium and 0.3 mm copper filters at a distance of 45 cm between the focus and the target. Radiation was carried out at 150 kV, 20 mA, 1.0 Gy/min. During X-ray exposure.

### Co-culture system

A transwell insert co-culture system was used to detect bystander effects. Exponentially growing HDFn cells (2 × 10^5^ cells) and 4 Gy exposed HDFn cells (2 × 10^5^ cells) were seeded in a 0.4 µm pore size insert (Corning Inc., Corning, USA) and HDFn cells (PDN8: 2 × 10^5^ cells) were seeded on collagen coated round glass coverslips (Corning Inc.) in the lower compartment of a 6-well transwell system, followed by incubation in humidified 5% CO_2_ incubator at 37 °C (Fig. S1C).

### Preparation of the sham or 4 Gy irradiated cell-conditioned medium

HDFn cells grown in T-25 flasks (1 × 10^6^ cells/flask) were cultured for 72 h in a humidified 5% CO_2_ incubator at 37 °C and 4 Gy irradiated HDFn cells in T25 flasks (1–2 × 10^6^ cells/flask) were cultured for 24, 48, and 72 h after X-ray exposure in a humidified 5% CO_2_ incubator at 37 °C. After cultivation, medium was collected from control or directly irradiated cells and filtered through a 0.22 µm filter. The control cell-conditioned medium (CCCM) or irradiated cell-conditioned medium (ICCM) were used for further experiments.

### Exosome-like vesicles (ELV) isolation and labellin**g**

ELV from the conditioned medium were isolated using the exoEasy Maxi Kit (Qiagen) according to the manufacturer’s instructions. ELV from mouse serum were collected after 72-h X-ray exposure using an ExoQuick Solution (System Biosciences). Briefly, blood was collected by cardiac puncture and following coagulation (1 h at room temperature), serum was separated via centrifugation at 10,000 g for 15 min using microtainer tubes (BD Biosciences) to remove cells and cellular fragments. Supernatants were filtered through a 0.45 μm pore polyvinylidene fluoride filter (Millipore). ExoQuick solution was added to the supernatants, and ELV were precipitated by refrigeration at 4 °C for 12 h. EV pellets were collected by centrifugation at 1500 g for 30 min and were dissolved in 20 μL phosphate-buffered saline (PBS). After collection of ELV from the medium or mouse serum, we hired a method for ELV purification by using Tim4 protein to obtain enriched ELV, which specifically binds the phosphatidylserine expressed on the surface of ELV^40^. The total protein content of ELV was determined using a Qubit 3.0 Fluorometer (Invitrogen) according to the manufacturer’s protocol. To study the ELV uptake, they were labelled with a fluorescent dye PKH-67 using the PKH-67 labeling kit (Sigma-Aldrich), according to manufacturer’s recommendations. The suspension was then filtered with a 100 kDa MW cut-off Amicon Ultra Concentrator (Millipore). HDFn or m5s cells were plated in 2-well chamber slides (1 × 10^5^ cells/well) and cultivated for 24 h. Slides were washed three times in PBS-, and medium with PKH67-labelled ELV or negative control (the flow-through) samples was added into each well (final concentration: 2 μg/mL). Cells were cultured for 24 h in a humidified 5% CO_2_ incubator at 37 °C. After incubation, the slides were washed three times in PBS- and fixed with 4% formaldehyde in PBS- for 15 min at room temperature. Fixed cells were washed extensively with PBS. Nuclear staining was counterstained with 4′, 6-diamino-2-phenylindole (DAPI; Sigma-Aldrich). Images were captured using a fluorescent microscope and CCD camera (Olympus).

### Protein detection by western blots

Western blot analysis was performed as described previously^41^. Cells or extracted ELV were washed once with PBS(−) and boiled for 10 min in 2 × Laemmli sample buffer (100 mM Tris-HCl, pH 6.8, 4% sodium dodecyl sulphate (SDS), 20% glycerol, 200 mM β-mercaptoethanol, 0.004% bromophenol blue) to promote complete lysis. Lysates were then electrophoresed on a 15% SDS-polyacrylamide gel and transferred onto a PVDF membrane (Bio-Rad). Non-specific sites were blocked with TBS-T (20 mM Tris-HCl, pH 7.8, 100 mM NaCl, 0.05% Tween-20) supplemented with 5% non-fat dry milk. Next, membranes were incubated overnight at 4 °C with primary antibodies against anti-CD9 (1:200; ab92726, Abcam), TSG101 (1:200; EPR7130(B), Abcam). Membranes were then washed three times with TBS-T for 10 min at room temperature before incubation for 2 h with a horseradish peroxidase (HRP)-linked anti-IgG secondary antibody (1:2000, goat anti-rabbit) (GE Healthcare). After three further TBS-T washes, membranes were developed using the ECL Prime Western Blotting Detection reagent as directed by the manufacturer (GE Healthcare). Chemiluminescence signals were assessed using a Lumicube (Liponics) with densitometric analysis performed using ImageJ software.

### Immunofluorescence staining

Immunofluorescent staining was performed as described previously^41^. Briefly, HDFn or m5s cells were plated in 2-well chamber slides (1 × 10^5^ cells/well) and cultured for 24 h. Cells were then washed three times in PBS- and cultured for 24 h in a medium containing 0.5 μg/mL ELV of CCCM or 0.5 μg/mL ELV of ICCM. Cells grown on a coverslip were washed three times in PBS- and then fixed with 4% formaldehyde in PBS- for 15 min at room temperature. After fixation, cells were washed extensively with PBS-, and incubated for 2 h at 37 °C with anti-γH2AX (1:100; 05-636, Millipore) and anti-53BP1 (1:80; ab36823, Abcam) antibodies in PBS-T supplemented with 5% non-fat dry milk. After three washes with PBS-, cells were incubated for 1 h at 37 °C with AlexaFluor488- and AlexaFluor648-conjugated secondary antibodies (1:500; Abcam). Nuclei were counterstained with DAPI.

### DNase, RNase, and protease treatment

ELV were purified from CCCM and ICCM, and then divided into four fractions. The first fraction was non-treated. The second, third, and fourth fractions were treated with RNase A (0.4 μg/μl), DNase (Promega), protease K (100 μg/ml), respectively, for 30 min in each case, and then each of the fractions was used to treat HDFn cells, as reported previously^42–44^.

### Generation of mitochondrial DNA depleted (Rho0) cell

HDFn cells were incubated in DMEM containing 10% FBS (Sigma-Aldrich), 1 mmol/L pyruvate, 50 mg/L uridine, and 100 g/L ethidium bromide to generate Rho0 cells. Cells were cultured in a humidified 5% CO_2_ incubator at 37 °C and the medium was changed every 2 days. Cells were cultured for 30–35 days and the generation of Rho0 cells was confirmed by PCR and RT-PCR using The Human Mitochondrial DNA (mtDNA) Monitoring Primer Set (Cat. #7246, Takara Bio Inc.) and data analysis was performed according to the manufacturer’s instructions. To visualize mitochondrial network in HDFn and Rho0 cells, cells were stained with MitoTracker CMXRos (Invitrogen) according to the manufacturer’s instructions.

### Quantification of mitochondrial DNA in exosome

For quantification of mitochondrial to nuclear DNA content in ELV, 50–100 ng of DNA was used and the protocol provided for the Human Mitochondrial DNA (mtDNA) Monitoring Primer Set (Takara Bio Inc.) was followed. For amplification of mouse mDNA (mND1 and mND5) and nDNA (mSLCO2B1 and mSEPIN3), validated mouse primer pairs for ND1; qMmuCEP0060079, ND5; qHsaCED0007714, SLCO2B1; qMmuCID0012408, SEPIN3; qMmuCID0012875 were obtained from PrimePCR^TM^ Assays (Bio-Rad). Real-time quantification to measure gene expression was performed using Power SYBR Green PCR Master Mix (Thermo Fisher Scientific) and compared the expression used human or mouse primers. The experiment was performed in quadruplicate.

### Exosome loading with mitochondrial DNA

Purified ELV derived from control HDFn were transfected with ND1, ND5, SLCO2B1, and SEPIN3 using Exo-Fect Exosome Transfection Kit (System Biosciences) according as per the manufacturer’s instructions. Following the same protocol, purified exosomes derived from control mouse serum were transfected with mND1, mND5, mSLCO2B1, and mSEPIN3. Briefly, PCR products were run on 1.5% agarose gel, cut at the desired length, and purified using Wizard SV Gel and PCR Clean-Up System (Promega). After purification of DNA, 50 μl of purified ELV PBS suspension were mixed with 10 μl of Exo-Fect solution and 20 μl of 0.2 μg of each amplified DNA. Transfected ELV were added to cells plated in 2-well chamber slides (1 × 10^5^ cells/well) and incubated for 24 h. Further, to observe the uptake of ELV, ELV containing amplified DNA were labelled with PKH-67 using the PKH-67 labeling kit according to manufacturer’s recommendations (Sigma-Aldrich).

### Statistical analysis

The statistical significance of the difference between groups was assessed using the statistical tests indicated in the figure legends. All statistical analyses were performed by using StatMate III software (ATMS). Statistical significance was set at *p* < 0.05 unless otherwise noted.

## Results

### Induction of DNA damage in cells treated with ELV from 4 Gy irradiated cells

**Figure 1:**
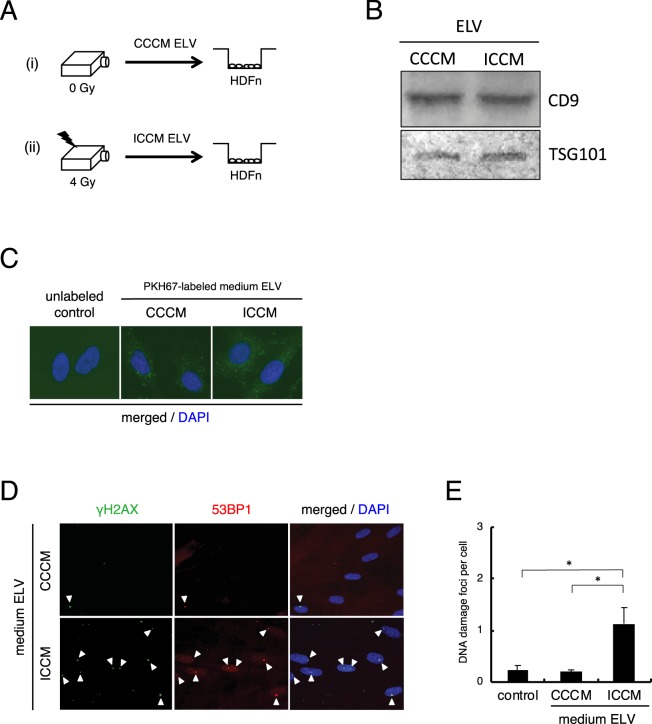
Induction of DNA damage by ELV obtained from irradiated cells. (**A**) A schematic view of the experimental protocol. (**B**) Detection of CD9 and TGS101 proteins in the exosome-like vesicles (ELV) in medium released from control HDFn cells (control cell-conditioned medium: CCCM) or 4 Gy exposed HDFn cells (irradiated cell-conditioned medium: ICCM) by Western blotting. Full-length blots are present in Supplementary Fig. S2. (**C**) Representative image of PKH67 labeled ELV (green) derived from CCCM or ICCM up-taken by treated HDFn cells, counterstained with 4′,6-diamidino-2-phenylindole (DAPI) which is blue. (**D**) Representative images of γH2AX (green) and 53BP1 (red) focus-positive cells treated with CCCM ELV or ICCM ELV treated HDFn cells, counterstained with DAPI. (**E**) The frequency of DNA damage foci in un-treated cells (control), cells treated with CCCM ELV and cells treated with ICCM ELV. Values are represented as mean ± standard error, with significant differences between indicated groups calculated (*) by Chi-square test (*p* < 0.01).

**Figure 2:**
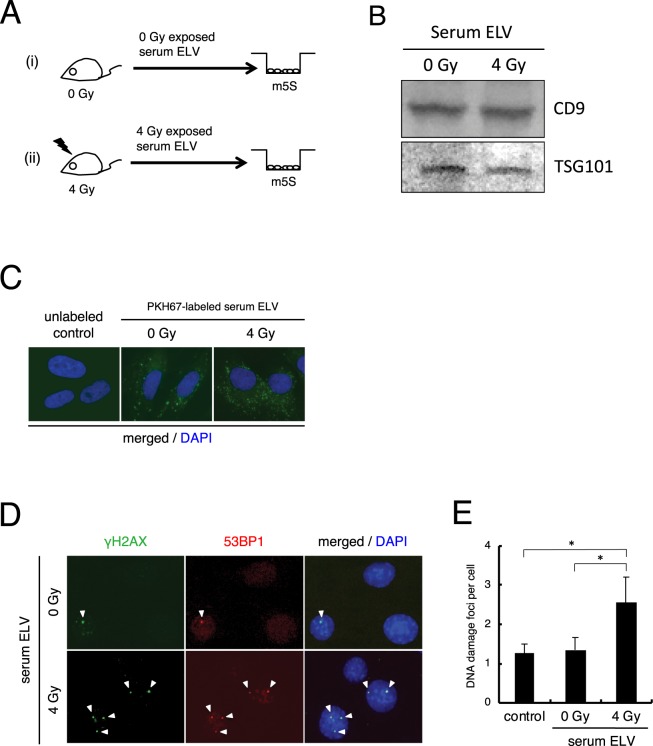
Induction of DNA damage by ELV obtained from irradiated mouse serum. (**A**) A schematic view of the experimental protocol. (**B**) Detection of CD9 and TGS101 proteins in the ELV in serum from control mouse or 4 Gy exposed mouse by Western blotting. Full-length blots are present in Supplementary Fig. S2. (**C**) Representative image of PKH67 labeled ELV (green) derived from control mouse serum or 4 Gy irradiated mouse serum up-taken by treated m5S cells, counterstained with DAPI (blue). (**D**) Representative images of γH2AX (green) and 53BP1 (red) focus-positive cells treated with un-irradiated mouse ELV or 4 Gy irradiated ELV, counterstained with DAPI. (**E**) The frequency of DNA damage foci in un-treated cells (control), cells treated with un-irradiated (0 Gy) mouse ELV or cells treated with irradiated (4 Gy) ELV. Values are represented as mean ± standard error (**E**), with significant differences between indicated groups (*) calculated by Chi-square test (*p* < 0.01).

We plated the exponentially growing HDFn cells in the bottom of the dish, and plated the sham-irradiated or 4 Gy irradiated HDFn cells to transwells to observe radiation-induced bystander effect (RIBE) in co-culture systems (Fig. S1A). To evaluate mutagenic response in the neighbouring cells of 4 Gy irradiated cells, we measured DNA damage in the neighbouring cells by staining for γH2AX and 53BP1 DNA repair foci after 24–72 h co-culture (Fig. S1B). The frequency of DNA damage foci in the HDFn control cells was 0.23 ± 0.09 and in the neighbouring sham-irradiated cells was 0.28 ± 0.08 (Fig. S1C). However, the frequency of DNA damage foci in the neighbouring 4 Gy irradiated cells had significantly increased after 24 h co-culture (1.26 ± 0.44) (*p* < 0.01), 48 h co-culture (1.66 ± 0.28) (*p* < 0.01) and 72 h co-culture (2.27 ± 0.51) (*p* < 0.01) (Fig. S1C). Next, we examined the effects of medium transfer of sham-irradiated control cell condition medium (CCCM) and 4 Gy irradiated cell condition medium (ICCM) after 72 h exposure (Fig. S1D). We observed a significant increase in DNA damage foci in ICCM treated cells (1.84 ± 0.61) compared to CCCM treated cells (0.24 ± 0.10) (*p* < 0.01) or non-treated control cells (0.23 ± 0.09) (*p* < 0.01) (Fig. S1E), suggesting that 4 Gy irradiated HDFn cells induced mutagenic response in neighbouring cells via culture media. Since the RIBE was observed in co-culturing systems and medium transfer experiments (Fig. S1), we next investigated the possibility of exosome-like vesicles (ELV) being involved in mediating RIBE (Fig. 1A). To obtain enriched ELV, we used a method for ELV purification by using Tim4 protein, which specifically binds to the phosphatidylserine displayed on the surface of ELV^40^. Seventy-two hours after sham- or 4 Gy exposure, CCCM or ICCM were collected and ELV were isolated and examined (Fig. 1A). Western blot analysis revealed a typical signature of proteins associated with ELV, such as CD9 and TSG101 (Fig. 1B). The amount of ELV from CCCM (CCCM ELV) and ELV from ICCM (ICCM ELV) was 0.51 ± 0.10 μg/ml and 0.49 ± 0.11μg/ml, respectively. So, we treated the bystander cells with final concentration 0.5 μg/ml ELV from CCCM or ICCM. Also, in all the following experiments, we set the final concentration of ELV as 0.5 μg/ml to treat bystander cells. In order to check the intake of ELV by the neighbouring cells, we stained the exosome with PKH67 green fluorescent reagent and observed the labeled ELV inside the cells after 6–12 hours treatment (Fig. 1C). Twenty-four hours after treatment with ELV, the frequency of DNA damage foci in cells treated with CCCM ELV (0.20 ± 0.04) was almost the same as non-treated cells (0.23 ± 0.09) (Fig. 2D,E). On the other hand, we observed significant increase in the frequency of DNA damage foci in cells treated with ICCM ELV (1.13 ± 0.31) (*p* < 0.01) as compared to that in non-treated cells or cells treated with CCCM ELV (Fig. 1D,E). These data suggest that ICCM ELV are involved in mediating RIBE.

### Induction of DNA damage in cells harvested with ELV from 4 Gy irradiated mouse serum

To examine RIBE in mouse models, we obtained serum from un-irradiated control mouse (8-week old, n = 3) and 4 Gy irradiated mouse (8-week old, n = 3), and collected ELV from each serum. We treated each ELV with cultured mouse fibroblast cells (m5S) (Fig. 2A). We first plated the growing m5S cells in the bottom of the dish and cultured cells in medium with ELV depleted FBS as control, un-irradiated (0 Gy) mouse serum ELV, and 4 Gy irradiated mouse serum ELV (Fig. 2A). Immunoblotting revealed a typical signature of proteins associated with ELV (CD9 and TSG101) (Fig. 2B). We stained each ELV with PKH67 green fluorescent reagent and treated them with m5S cells, and observed the labeled ELV inside the treated cells after 6 hours of treatment (Fig. 2C). Twenty-four hours after treatment, the frequency of cells positive for DNA damage foci after treatment with the control mouse serum ELV (1.35 ± 0.32) was almost the same as that in non-treated control m5S cells (1.27 ± 0.23) (Fig. 2D,E). The frequency of DNA damage foci in non-treated control m5S (1.27 ± 0.23) were higher than non-treated control HDFn (0.23 ± 0.09), and it have been reported that cells in the S-phase with undamaged DNA display γH2AX constitutively^45^, also the number of foci in the G2-phase are higher than in the G1-phase^46^. So we analyzed the distribution of cell cycle of each cells and found that the population of cells in S and G2/M phase of m5S cells (G1: 36.0 ± 1.51%, S: 17.4 ± 1.12%, G2/M: 28.2 ± 1.59%) were higher than that of HDFn cells (G1: 62.0 ± 0.4%, S: 13.4 ± 0.81%, G2/M: 17.5 ± 0.71%). These results suggest that cell cycle could affect the background foci numbers. Compared to non-treated control m5S cells (1.27 ± 0.23) or control mouse serum ELV (1.35 ± 0.32), ELV of 4 Gy irradiated mouse serum treatment induced DNA damage foci (2.55 ± 0.66) in m5S cells (Fig. 2D,E). These results suggest that ELV from 4 Gy irradiated mouse serum induce RIBE.

### DNA in ELV of 4 Gy irradiated cells induce DNA damage

**Figure 3:**
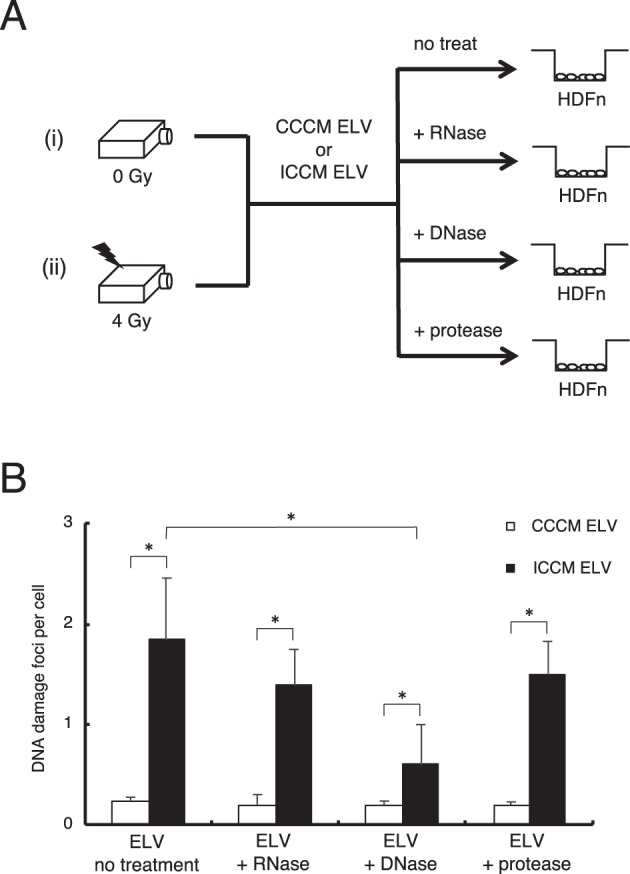
Effect of ICCM treated with RNases, DNases, and proteases on CCCM ELV and ICCM ELV. (**A**) A schematic view of the experimental protocol. (**B**) The frequency of DNA damage foci in cells treated with CCCM ELV and ICCM ELV (un-treated), cells treated with RNase exposed CCCM ELV and ICCM ELV (+RNase), cells treated with DNase exposed CCCM ELV and ICCM ELV (+DNase), and cells treated with protease exposed CCCM ELV or ICCM ELV (+protease). Values are represented as mean ± standard error, with significant differences between indicated groups (*) calculated by Chi-square test (*p* < 0.01).

It has been suggested that ELV are important mediators of intercellular communication through their contents such as mRNAs, miRNAs, DNA, and proteins^23^. To investigate which component is involved in the induction of RIBE, we examined the induction of DNA damage in cells treated with CCCM ELV and ICCM ELV (un-treated ELV), CCCM ELV and ICCM ELV pre-treated with RNase (ELV + RNase), CCCM ELV and ICCM ELV pre-treated with DNase (ELV + DNase), and CCCM ELV and ICCM ELV pre-treated with proteinase K (ELV + proteinase) (Fig. 3A). We did not find significant differences in DNA damage foci between CCCM ELV + RNase (0.20 ± 0.10), CCCM ELV + DNase (0.19 ± 0.03), CCCM ELV + proteinase (0.20 ± 0.03), and un-treated ELV (0.23 ± 0.04) (Fig. 3B). While we could not observe significant differences in DNA damage foci between ICCM ELV + RNase (1.01 ± 0.31) or ICCM ELV + proteinase (1.00 ± 0.38) as compared to that in un-treated ELV (1.27 ± 0.44) (Fig. 3B), ICCM ELV + DNase significantly reduced DNA damage foci (0.39 ± 0.28) (*p* < 0.01) as compared to that of un-treated ELV (Fig. 3B). It must be noted that DNA damage in cells treated with ICCM ELV + DNase (0.39 ± 0.28) was significantly higher than CCCM ELV + DNase (0.19 ± 0.03). These results suggest that DNA in ICCM ELV is involved in transducing RIBE signals, also other component is involved in the induction of RIBE.

### No induction of DNA damage in cells treated with exosomes of rho 0 cells

**Figure 4:**
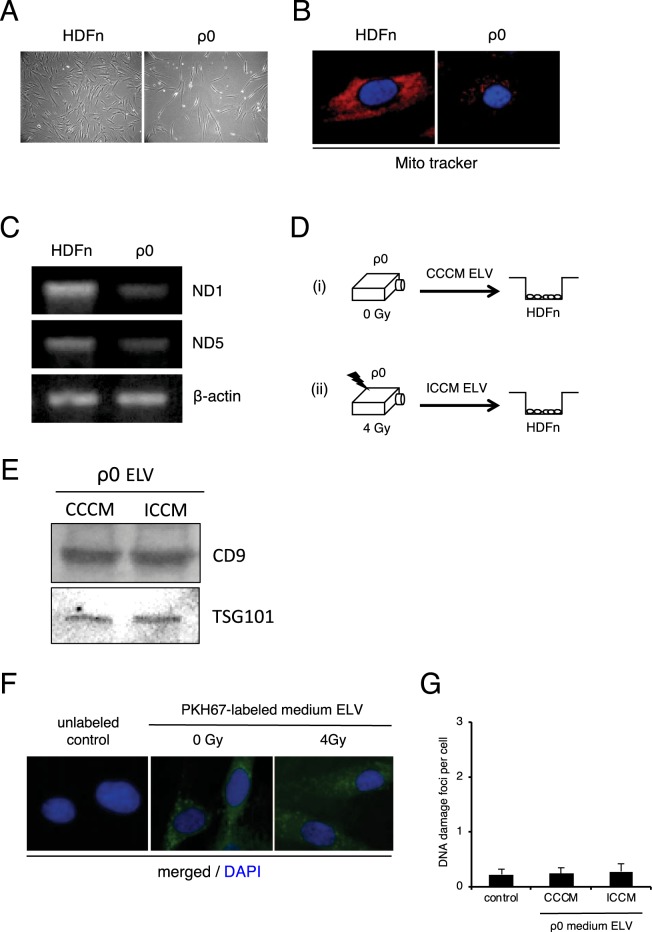
No induction of DNA damage by ELV obtained from mitochondria depleted (rho 0) cells. (**A**) Morphology of rho 0 (ρ0) cells and their parental (HDFn) cells. (**B**) Representative image of MitoTracker CMXRos retention (red) in HDFn cells and ρ0 cells, counterstained with DAPI (blue). (**C**) PCR amplification of mitochondrial ND1 (upper panel) and ND5 (middle panel), internal control beta-actin (lower panel) of HDFn cells and ρ0 cells. Full images of gels are present in Supplementary Fig. S3. (**D**) A schematic view of the experimental protocol. (**E**) Detection of CD9 and TGS101 proteins in the ELV in medium from control ρ0 cells and 4 Gy exposed ρ0 cells. Full-length blots are present in Supplementary Fig. S2. (**F**) Representative image of PKH67 labeled ELV (green) derived from control ρ0 cells and 4 Gy exposed ρ0 cells, counterstained with DAPI (blue). (**G**) The frequency of DNA damage foci in un-treated cells (control), cells treated with ρ0 CCCM ELV and cells treated with ρ0 ICCM ELV. Values are represented as mean ± standard error, with significant differences between indicated groups (*) calculated by Chi-square test (*p* < 0.01).

As reported previously, mitochondria depleted ρ_0_ (rho 0) cells do not exhibit RIBE^47,48^. We generated ρ_0_ cells and evaluated the mutagenic response of ELV from ρ_0_ cells. To obtain ρ_0_ cells, we cultured the HDFn cells in medium containing ethidium bromide (EtBr) for 15–20 days, and observed the cells which showed morphological differences as compared to the parental cells (Fig. 4A). To assess the mitochondrial function, we stained the cells with MitoTracker Red CMXRos (Fig. 4B) and PCR analysis of mitochondrial DNA (ND1 and ND5) in ρ_0_ cells and paternal HDFn cells was performed (Fig. 4C). We observed the decrease in MitoTracker Red CMXRos signal intensity in ρ_0_ cells (Fig. 4B) and the intensity of the bands of ND1 and ND5 indicated that mitochondrial amount is much lower in ρ_0_ cells as compared to the paternal HDFn cells (Fig. 4C). To determine whether ELV from ρ_0_ cells induced RIBE, we investigated the induction of DNA damage in cells which were treated with ELV from CCCM of ρ_0_ cells and ICCM of ρ_0_ (4 Gy irradiated) cells (Fig. 4D). We again examined the immunoblot analysis of ELV (CD9 and TSG101) (Fig. 4C) and stained the ELV from ρ_0_ cells with PKH67 green fluorescent reagent and observed the labeled exosomes inside the treated cells after 6 hours of treatment (Fig. 4F). After 24 hours treatment with ELV, the frequency of cells positive for DNA damage foci in cells treated with CCCM ELV of ρ_0_ (0.24 ± 0.11) and cells treated with ICCM ELV of ρ_0_ (0.28 ± 0.14) was almost the same as that in non-treated control cells (0.23 ± 0.09) (Fig. 4G). These results suggest that ELV from ρ_0_ cells do not induce RIBE signal.

### Mitochondrial DNA in ELV

**Figure 5:**
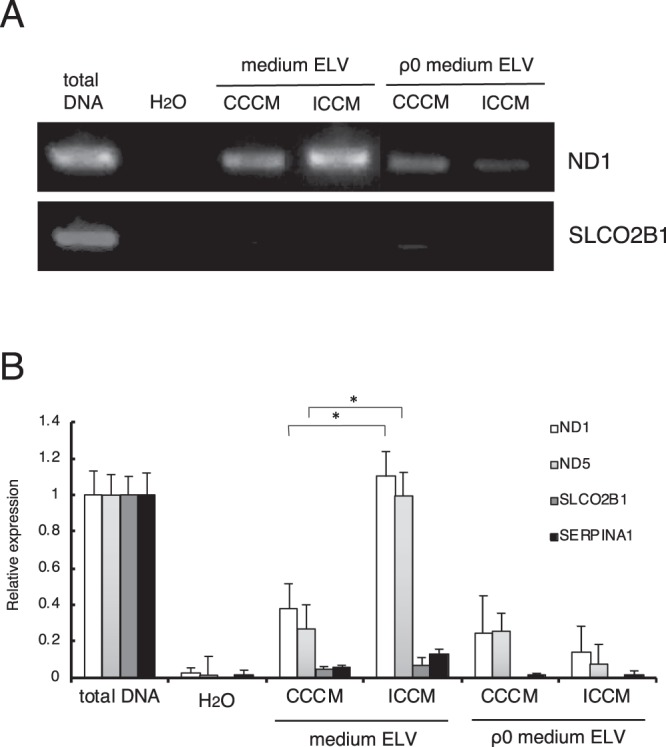
Mitochondrial DNA in ELV. (**A**) PCR amplification of mitochondrial ND1 (upper panel) and nuclear SLCO2B1 (lower panel) in CCCM ELV, ICCM ELV, ρ0 CCCM ELV, and ρ0 ICCM ELV. Full images of gels are present in Supplementary Fig. S3. (**B**) Quantification of mitochondrial DNA (mtDNA; ND1 and ND5) and nuclear DNA (nDNA; SLCO2B1 and SERPINA1) by RT-PCR in CCCM ELV, ICCM ELV, ρ0 CCCM ELV, and ρ0 ICCM ELV. Values are represented as mean ± standard error, with significant differences between indicated groups (*) calculated by Chi-square test (*p* < 0.01).

Although, mtDNA in ELV has not been reported to mediate RIBE signals, several studies suggest that ELV play crucial roles in mediating RIBE signals^31,34,49^ possibly via RNA and protein^32^, or via miR-7-5p^34^. In our data, RIBE was not observed after treatment with ICCM ELV from ρ_0_ (Fig. 4G). The fact that DNA in ICCM ELV is transducing RIBE signals (Fig. 3B) imply that mitochondrial component, especially mtDNA in ICCM ELV may mediate RIBE signals. To test this hypothesis, we investigated the quantification of mtDNA in ELV by PCR (Fig. 5A) and RT-PCR (Fig. 5B). After reaction with endogenous gene primes (SLCO2B1 and SERPINA1), very few endogenous genes were observed in CCCM ELV, ICCCM ELV of HDFn cells, or in CCCM ELV, and ICCCM ELV of ρ_0_ cells (Fig. 5A,B). However, after treatment with mitochondrial gene primers (ND1 and ND5), higher amount of mtDNA was observed in ICCM ELV as compared to CCCM ELV of HDFn cells (Fig. 4A,B). Very low amount of mtDNA was detected in CCCM ELV and ICCM ELV from ρ_0_ cells (Fig. 4A,B). It is notable that mtDNA in CCCM ELV of ρ_0_ cells was lower than CCCM ELV of HDFn cells, also the amount did not change in ICCM ELV of ρ_0_ cells (Fig. 5A,B).

### Exosome loading with mitochondrial DNA induces DNA damage

**Figure 6:**
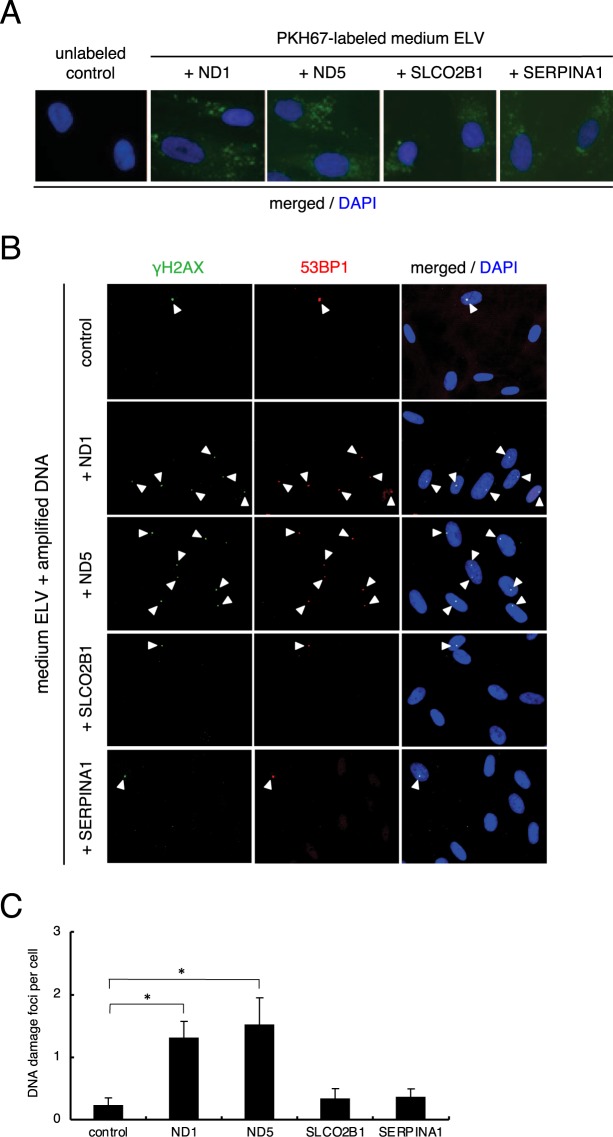
Induction of DNA damage by amplified human mtDNA in ELV. (**A**) Representative image of PKH67 labeled ELV (green) derived from CCCM with amplified mtDNA (+ND1 and +ND5) or with amplified nDNA (+SLCO2B1 and + SERPINA1) up-taken by treated HDFn cells, counterstained with DAPI (blue). (**B**) Representative images of γH2AX (green) and 53BP1 (red) focus-positive cells in un-treated cells (control), CCCM ELV + ND1, CCCM ELV + ND5, CCCM ELV + SLCO2B1, and CCCM ELV + SERPINA1 treated cells, counterstained with DAPI. (**C**) The frequency of DNA damage foci in un-treated cells (control), CCCM ELV + ND1, CCCM ELV + ND5, CCCM ELV + SLCO2B1, and CCCM ELV + SERPINA1 treated cells. Values are represented as mean ± standard error, with significant differences between indicated groups (*) calculated by Chi-square test (*p* < 0.01).

**Figure 7:**
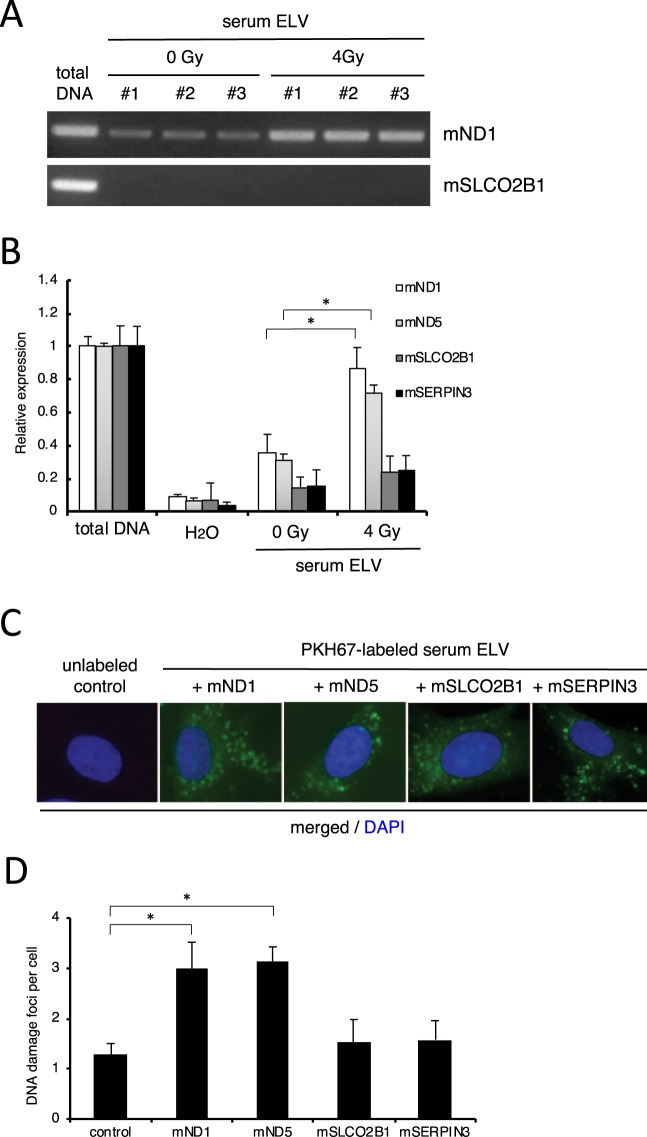
Induction of DNA damage by amplified mouse mtDNA in ELV. (**A**) PCR amplification of mitochondrial mND1 (upper panel) and nuclear mSLCO2B1 (lower panel) in control (0 Gy) mouse serum ELV (n = 3; #1 - #3) and irradiated (4 Gy) mouse serum ELV (n = 3; #1 - #3). Full images of gels are present in Supplementary Fig. S3. (**B**) Quantification of mitochondrial DNA (mtDNA; mND1 and mND5) and nuclear DNA (nDNA; mSLCO2B1 and mSERPINA1) by RT-PCR in control (0 Gy) mouse serum ELV and irradiated mouse serum ELV. Values are represented as mean ± standard error, with significant differences between indicated groups (*) calculated by Chi-square test (*p* < 0.01). (**C**) Representative image of PKH67 labeled ELV (green) derived from control mouse serum ELV with amplified mtDNA (+ND1 and +ND5) and amplified nDNA (+SLCO2B1 and + SERPINA1) up-taken by treated m5S cells, counterstained with DAPI (blue). (**D**) The frequency of DNA damage foci in un-treated cells (control), control mouse serum ELV + ND1, control mouse serum ELV + ND5, control mouse serum ELV + SLCO2B1, and control mouse serum ELV + SERPINA1 treated cells. Values are represented as mean ± standard error, with significant differences between indicated groups (*) calculated by Chi-square test (*p* < 0.01).

To investigate the physiological impact of mtDNA in ICCM ELV, we introduced the amplified mtDNA sequence (ND1 and ND5), or endogenous DNA sequence (SLCO2B1 and SERPINA1) as a control, into CCCM ELV by using Exofect reagent (see Material and Methods). The length of the amplified sequences of ND1, ND5, SLCO2B1, and SERPINA1 were almost the same (150–200 bp). After the introduction of ND1, ND5, SLCO2B1, and SERPINA1, we stained each ELV with PKH67 green fluorescent reagent and observed the labeled exosomes inside the treated cells after 6 hours of treatment (Fig. 6A). Twenty-four hours after treatment with ELV, the frequency of cells positive for DNA damage foci which contained amplified SLCO2B1 (+SLCO2B1) (0.35 ± 0.15) and amplified SERPINA1 (+SERPINA1) (0.35 ± 0.14) were almost the same as that of control (CCCM ELV of HDFn) (0.23 ± 0.11) (Fig. 6B,C). However, we observed a significant increase in DNA damage foci in cells treated with ELV which contained amplified ND1 (+ND1) (1.30 ± 0.27) and amplified ND5 (+ND5) (1.52 ± 0.42) as compared to the control (Fig. 6C). Next, we investigated mtDNA in ELV of mouse serum by PCR and RT-PCR (Fig. 7A). After treatment with endogenous gene primes (mSLCO2B1 and mSERPIN3) and mtDNA primers (mND1 and mND5), very few endogenous genes were observed in sham-irradiated mouse serum ELV (n = 3) and 4 Gy irradiated mouse serum (n = 3) (Fig. 7A). On the other hand, higher amount of mND1 or mND5 was observed in 4 Gy irradiated mouse serum ELV as compared to that in sham-irradiated mouse serum ELV (Fig. 7B). We also introduced amplified mtDNA or amplified endogenous DNA into sham-irradiated mouse ELV and then treated them with m5S cells (Fig. 7C,D). ELV labeled with PKH67 were observed after 6 hours of treatment (Fig. 7C), and after 24 hours of treatment, the frequency of cells positive for DNA damage foci in cells treated with ELV which contained amplified mSLCO2B1 (+mSLCO2B1) (1.53 ± 0.45) and amplified mSERPIN3 (+mSERPIN3) (1.58 ± 0.38) were almost the same as sham-irradiated control ELV (1.27 ± 0.23) (Fig. 6B,C). We observed a significant increase in DNA damage foci in cells treated with ELV which contained amplified mND1 (+mND1) (2.98 ± 0.54) and amplified mND5 (+mND5) (3.14 ± 0.29) as compared to the control (Fig. 7D). These results suggest that mtDNA in ELV is involved in transducing RIBE signal via culture medium or mouse serum.

## Discussion

In the present study, we found that the amount of mtDNA in ELV increased after radiation exposure in normal human fibroblast cells and in mouse serum. ELV from mitochondria depleted cells showed no increase in mtDNA after radiation exposure. ELV carrying more amount of partial mitochondrial DNA after radiation exposure induced DNA damage in treated cells, and amplified partial mtDNA induced DNA damage in treated cells.

When RIBE is manifested by a release of cytokines, a COX-2-dependent pathway mediated by FasL and TNF-α^50,51^, releasing NO by irradiated cells, triggers the inflammatory response in un-irradiated cells through the p38 pathway^52^. Additionally, several studies have demonstrated that ELV are involved in mediating RIBE in cultured cells^31–34,37–39^ and under *in vivo* conditions^35,36^. These studies suggest that inflammatory responses are involved in mediating RIBE signals.

Interestingly, accumulating evidences have suggested that extracellular mtDNA also induces inflammatory responses. Borghini *et al*. reported that increased serum cell free mitochondrial DNA fragments in interventional cardiologists are exposed to chronic low-dose radiation^53^. Collins *et al*. demonstrated that synovial fluids in rheumatoid arthritis patients contained mtDNA; mtDNA with oxidatively damaged base induced arthritis in mouse models^54^. Additionally, it was reported that hypomethylated CpG motifs of mtDNA activate the human neutrophils via TLR9^55^. Further, mtDNA that escaped from autophagy led to TLR 9-mediated inflammatory responses in cardiomyocytes, myocarditis, and dilated cardiomyopathy^56^. Guestini *et al*. first reported that glioblastoma cells and astrocytes release ELV carrying mtDNA^57^, and a higher level of mtDNA in ELV was observed in patients of chronic heart failure as compared to the healthy donors; mtDNA induced inflammatory response through the TLR9-NF-κB pathway^58^. Notably, Szczesny *et al*. reported that low levels of ROS, released into the extracellular environment via ELV, induce damage to mtDNA and activate inflammation via the Z-DNA binding protein 1 (ZBP1)^59^. Damaged mtDNA in ELV is capable of inducing inflammation in naïve pulmonary epithelial cells^59^. In relation to radiation, mitochondria is one of the major targets of radiation^60^. Radiation exposure causes mitochondrial dysfunction and increases the mitochondrial number^60,61^. Hence, Murphy *et al*. reported that direct radiation and ICCM both can induce point mutation and deletion type of mutation in mtDNA^62^. In addition, radiation can induce mitochondrial fission^63–65^, which has been proposed to activate mitophagy, the mechanism involved in the elimination of damaged mitochondria^66^. Thus, mtDNA damaged by direct radiation or ICCM might be eliminated via mitophagy, but it is possible that some portion of damaged mtDNA is extruded via exosomes by activation of the ZBP1 pathway.

Mitochondria possess their own genome, which being a circular DNA molecule and with nonmethylated CpG motifs resembles bacterial DNA^67^. Therefore, it is agreeable that CpG of mtDNA activates neutrophils via CpG/TLR9 interaction^55^. In our experiment, induction of RIBE by ELV containing ND1 or ND5 in HDFn (Fig. 6) and mND1 or mND5 in m5S cells were observed (Fig. 7). According to Lie *et al*., CpG methylation in mtDNA is a rare event at most DNA regions^68^, suggesting that nonmethylated CpG motifs of ND1, ND5, mND1, and mND5 in ELV could induce inflammatory response. Additionally, it is notable that RIBE (elevated level of DNA double-stranded breaks) elicited by extracellular genomic DNA from irradiated cells was reduced after inhibition of TLR9^69^, suggesting that innate immune response mediated by TLR9 contributes to the induction of retinoic acid-inducible gene-I (*RIGI*).

Several studies have revealed that mitochondria depleted cells (Rho0 cells) do not produce RIBE signals^47,48,70^ and our data also suggests that ICCM ELV from Rho0 cells could not induce DNA damage in treated cells (Fig. 4). These studies focused on the ROS or reactive nitrogen species produced by functional mitochondria^47,48,70^. In our experiments, ELV from Rho0 cells showed no induction of DNA damage in treated cells (Fig. 4G), and ELV from Rho0 cells contained small amount of ND1 and ND5 (Fig. 5), which could be the reason for the absence of RIBE signals. Taken together, these results demonstrated the residual effect of DNA damage induction of DNase treated ICCM ELV (Fig. 3B). These lines of evidences suggest that ROS or other factors also mediate the RIBE signals, as observed in our experiments.

In summary, present study suggests that RIBE may be partially mediated by mtDNA in ELV.

## Supplementary information

Supplementary information
