# Supplementary material for: Radiation-Induced Bystander Effect is Mediated by Mitochondrial DNA in Exosome-Like Vesicles
Source: Sci Rep. 2019 Jun 24;9:9103. doi: 10.1038/s41598-019-45669-z (PMC6591216; doi:10.1038/s41598-019-45669-z)
Supplement: Supplementary file 1 — Supplementary information [file 41598_2019_45669_MOESM1_ESM.docx]

**Supplementary information**

**Radiation-Induced Bystander Effect is Mediated by Mitochondrial DNA in Exosome-Like Vesicles**

Kentaro Ariyoshi^1,^*, Tomisato Miura^2^, Kosuke Kasai^2^, Yohei Fujishima^2^, Akifumi Nakata^3^, Mitsuaki Yoshida^1,^*

^1^Department of Radiation Biology, Institute of Radiation Emergency Medicine, Hirosaki University, 66-1 Hon-cho, Hirosaki 036-8564, Japan

^2^Department of Biomedical Sciences, Hirosaki University Graduate School of Health Sciences, 66-1 Hon-cho, Hirosaki 036-8564, Japan

^3^ Department of Basic Pharmacy, Hokkaido Pharmaceutical University School of Pharmacy, 7-1 Katsuraoka-cho, Otaru, Hokkaido 047-0264, Japan

***Corresponding authors:**

Kentaro Ariyoshi, Ph.D. and Mitsuaki A. Yoshida, Ph.D.

Hirosaki University, Institute of Radiation Emergency Medicine, 66-1 Hon-cho, Hirosaki 036-8564, Japan.

Tel: +81-172-39-5931

FAX: +81-172-39-5931

E-mail addresses: [ariyoshi@hirosaki-u.ac.jp](mailto:ariyoshi@hirosaki-u.ac.jp); [myoshida@hirosaki-u.ac.jp](mailto:myoshida@hirosaki-u.ac.jp)


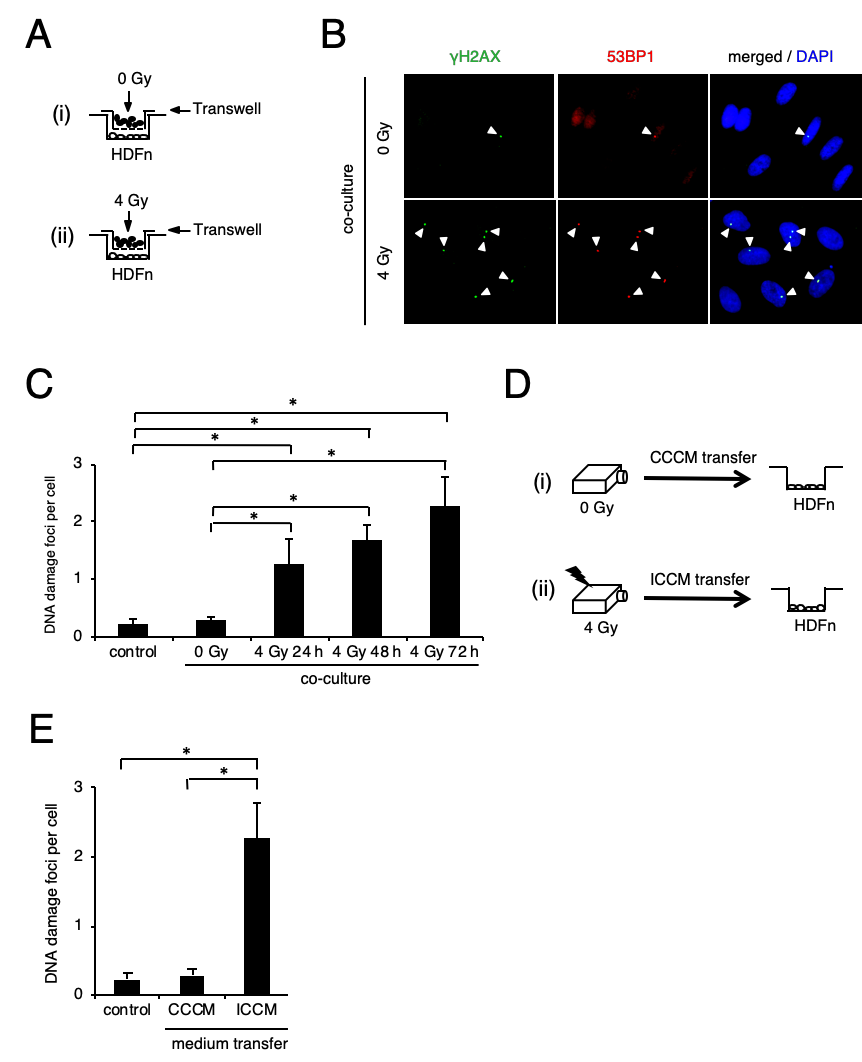
**Fig. S1. Induction of DNA damage in bystander cells mediated by the medium.**

(A) A schematic view of the experimental protocol of co-culture. (B) Representative images of γH2AX (green) and 53BP1 (red) focus-positive cells in HDFn cells with co-cultured control (0 Gy) HDFn cells or irradiated (4 Gy) HDFn cells for 72 h. (C) The frequency of DNA damage foci in HDFn cells (control), HDFn co-cultured with control (0 Gy) HDFn cells for 72h, HDFn co-cultured with irradiated (4 Gy) HDFn cells for 24 h, HDFn co-cultured with irradiated (4 Gy) HDFn cells for 48 h and HDFn co-cultured with irradiated (4 Gy) HDFn cells for 72 h. (D) A schematic view of the experimental protocol of the medium transfer. (E) The frequency of DNA damage foci in un-treated cells (control), CCCM treated cells, and ICCM treated cells. Values are represented as mean ± standard error, with significant differences between indicated groups (*) calculated by Chi-square test (*p*<0.01).

**Fig. S2. The Full-length blots.**

Detection of CD9 (A), and TSG101 (B) proteins in CCCM ELV or ICCM ELV released from HDFn, ELV in serum from control mouse or 4 Gy exposed mouse, CCCM ELV or ICCM ELV from ρ0 cells. ELV from CCCM or ICCM of HDFn were isolated using an ExoQuick (SBI) or the exoEasy Maxi Kit (Qiagen) according to the manufacturer’s instructions. After isolation by exoEasy Maxi kit, ELV purification by using Tim4 protein to obtain enriched ELV.

**Fig. S3. The Full-images of gels.**

(A) PCR amplification of mitochondrial ND1 and ND5, internal control beta-actin of HDFn cells and ρ0 cells. (B) PCR amplification of mitochondrial ND1 and nuclear SLCO2B1 in CCCM ELV, ICCM ELV, ρ0 CCCM ELV, and ρ0 ICCM ELV. And mitochondrial mND1 and nuclear mSLCO2B1 in control (0 Gy) mouse serum ELV (n=3; #1 - #3) and irradiated (4 Gy) mouse serum ELV (n=3; #1 - #3) (C).
